# Structural characterization of a neutralizing mAb H16.001, a potent candidate for a common potency assay for various HPV16 VLPs

**DOI:** 10.1038/s41541-020-00236-w

**Published:** 2020-09-23

**Authors:** Weijin Huang, Maozhou He, Tingting Ning, Jianhui Nie, Feng Zhang, Qingbing Zheng, Rui Zhang, Ying Xu, Ying Gu, Shaowei Li, Youchun Wang

**Affiliations:** 1 Division of HIV/AIDS and Sexually Transmitted Virus Vaccines, National Institutes for Food and Drug Control (NIFDC), No.31 Huatuo Street, Daxing District, 102629 Beijing, China; 2 State Key Laboratory of Molecular Vaccinology and Molecular Diagnostics, School of Life Sciences, Xiamen University, 361102 Xiamen, China; 3 Department of Gastroenterology, Beijing Friendship Hospital, Capital Medical University, National Clinical Research Center for Digestive Disease, Beijing Digestive Disease Center, Beijing Key Laboratory for Precancerous Lesion of Digestive Disease, 100050 Beijing, China; 4 Division of Monoclonal Antibodies, National Institutes for Food and Drug Control (NIFDC), No.31 Huatuo Street, Daxing District, 102629 Beijing, China; 5 GE Healthcare Life Sciences, No.1 Tongji South Road, Yizhuang Business Development Area, 100176 Beijing, China

**Keywords:** Biotechnology, Immunology

## Abstract

With more human papillomavirus (HPV) virus-like particle (VLP) vaccines to hit the market in future, a monoclonal antibody (mAb) with preferably comparable reactivity against vaccines from different expression systems and bioprocesses is urgently needed for the potency characterization. Among all mAbs against HPV16 collected, rabbit mAb H16.001 is potently neutralizing with the highest affinity, recognizes an immune-dominant epitope, and can comparably react with HPV16 vaccines from various sources. Cryo-electron microscopic (cryo-EM) structure demonstrated that 360 H16.001 Fabs could bind to HPV16 capsid in preferable binding manner without steric hindrance between neighboring Fabs, potentially supporting its identification for VLP structural integrity and utility in monitoring VLP structural probity. This structural analysis indicated that mAb H16.001 afforded unbiased potency characterization for various HPV16 vaccines and was potential for use in vaccine regulation practice. This study also showed a model process for selecting suitable mAbs for potency assays of other vaccines.

## Introduction

Cervical cancer is the fourth most common female malignancy worldwide and represents a major global health challenge^1^. Among the human papillomavirus (HPV) types associated with this carcinoma, HPV16 is the most prevalent genotype and accounts for 50% of tumor specimens^2^.

The major capsid protein L1 of HPV can self-assemble into empty virus-like particles (VLPs) that are virion sized, highly immunogenic, and therefore the immunogens in several HPV vaccines, including Cervarix, Gardasil, Gardasil 9, and Cecolin^3–7^. These HPV vaccines conferred high protection efficacy against CIN2+ symptom in clinical trials and the protection is believed to be mediated by L1-neutralizing antibodies, which can be detected in the sera and cervicovaginal secretions of naturally infected or vaccinated individuals^8,9^.

Potency characterization is one of the most critical items in the quality control (QC) and regulatory lot release for HPV vaccines. Vaccine potency assay is usually conducted in vivo by an ED50 (half of effective dosage) value associated with neutralizing antibody elicitation in experimental mouse or alternatively measured in vitro by a neutralizing monoclonal antibody (mAb)-based double-antibody sandwich enzyme-linked immunosorbent assay (ELISA), also referred as in vitro relative potency (IVRP) that is well established with significant correlation to ED50 test. However, it is a challenge to develop a common IVRP test across various HPV vaccines, VLP antigens of which are produced in different expression systems, such as yeast, baculovirus-based insect cell and bacterial, or distinct manufacturing processes and therefore require an mAb that should recognize immunodominant neutralizing epitope and equally react with all sorts of VLPs.

For the purpose of finding an optimal antibody, a panel of mAbs against HPV16 L1 VLPs was characterized by a variety of immune assays. The rabbit mAb H16.001 was the optimal candidate for the assessment of the epitope integrity and antigenicity of VLP vaccine products, in the virtue of its potent neutralizing activity, high affinity in surface plasma resonance (SPR), and comparable reactivity against VLPs derived from different expression systems. An atomic model of HPV16 pseudovirus (PsV) in complex with H16.001 was obtained to demonstrate a unique binding preference for the IVRP assay. This study provides insights for selecting mAb used for a QC reagent for regulatory approval that may cover different manufacturing process and different expression systems.

## Results

### Neutralization profile of a panel of HPV16 mAbs

**Fig. 1:**
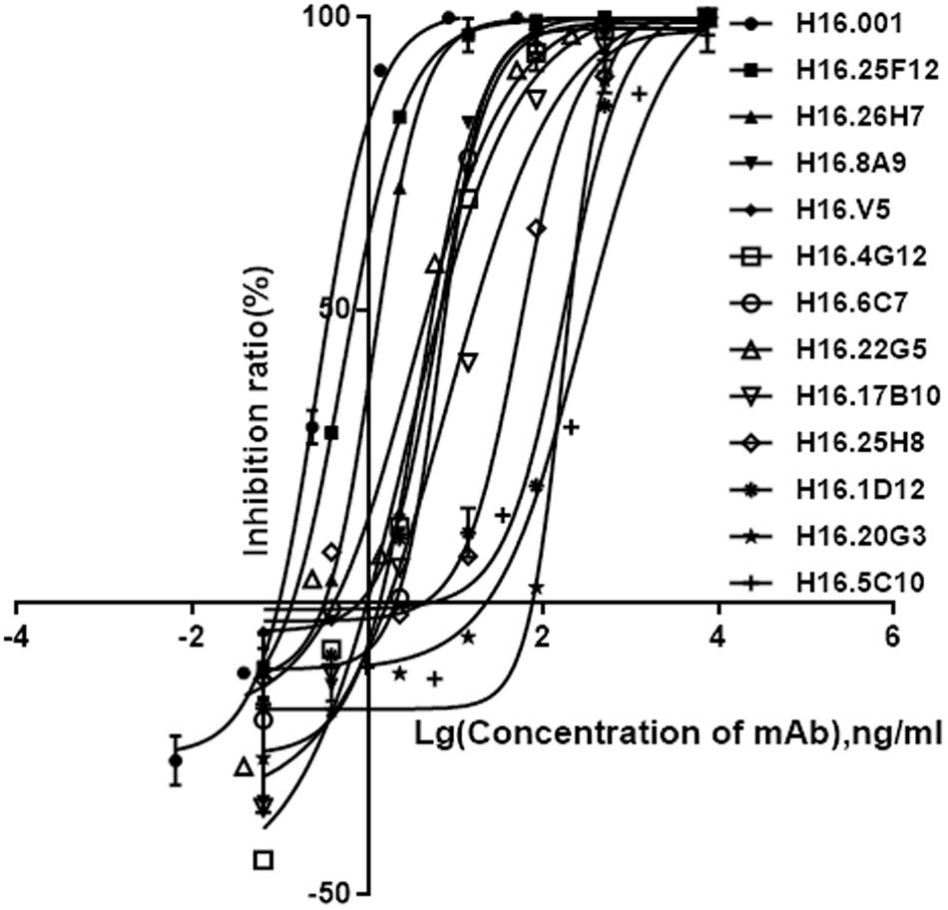
Neutralizing activities of the 13 mAbs. IC_50_ neutralizing activity was defined as the antibody concentration required to block viral entry by 50%. The inhibition ratios are indicated on the *Y*-axis, and antibody concentrations are indicated on the *X*-axis. Each data point is the mean of two separate experiments.

To establish a common neutralizing mAb (nAb)-based characterization approach to HPV16 vaccines generating from various expression systems, we first measured the in vitro neutralizing activities of a collection of 13 HPV16 mAbs (H16.001, H16.25F12, H16.26H7, H16.4G12, H16.V5, H16.8A9, H16.17B10, H16.6C7, H16.22G5, H16.25H8, H16.20G3, H16.1D12, and H16.5C10) by a PsV-based neutralization assay. The tests were conducted at a starting mAb concentration of 7.5 μg/mL, subsequent twofold serial dilution and inhibition ratio curves were generated for the 50% maximal inhibitory concentration (IC_50_) calculation. Six mAbs—H16.001, H16.25F12, H16.26H7, H16.8A9, H16.V5, and H16.4G12—showed excellent neutralizing activities with IC_50_ values of 0.40, 0.81, 1.29, 6.17, 6.77, and 7.52 ng/mL (Table 1 and Fig. 1), which were chosen for further study, while the rest 7 mAbs, including H16.6C7, H16.22G5, H16.17B10, H16.25H8, H16.1D12, H16.20G3, and H16.5C10, exhibited lower neutralizing activities with IC_50_ values of 7.62, 10.53, 20.12, 53.45, 192.30, 220.22, and 392.19 ng/mL. Notably, H16.001 had the most potent neutralizing activity, with 18-fold higher than that of the well-known mAb H16.V5^10,11^.

**Table 1:** Neutralizing activities of the 13 mAbs.

Neutralizing mAbs	Donator (vaccine manufacturers)	Species	Epitope	IC_50_ (ng/mL)
H16.001	Sino Biological Inc.	Rabbit	Conformational	0.40 (0.33–0.42)
H16.25F12	Beijing Health Guard Biotechnology	Mouse	Conformational	0.81 (0.62–0.86)
H16.26H7	Beijing Health Guard Biotechnology	Mouse	Conformational	1.29 (1.01–1.34)
H16.8A9	Innovax Biotech	Mouse	Conformational	6.17 (5.67–8.99)
H16.V5	Bowei Biotechnology	Mouse	Conformational	6.77 (5.18–7.59)
H16.4G12	Beijing Health Guard Biotechnology	Mouse	Conformational	7.52 (0.89–8.77)
H16.6C7	Beijing Health Guard Biotechnology	Mouse	Conformational	7.62 (4.04–11.3)
H16.22G5	Beijing Health Guard Biotechnology	Mouse	Conformational	10.53 (4.13–12.83)
H16.17B10	Beijing Health Guard Biotechnology	Mouse	Conformational	20.12 (11.48–21.66)
H16.25H8	Beijing Health Guard Biotechnology	Mouse	Conformational	53.45 (28.31–104.30)
H16.1D12	Innovax Biotech Co., Ltd	Mouse	Conformational	192.30 (56.52–557.90)
H16.20G3	Beijing Health Guard Biotechnology	Mouse	Conformational	220.22 (87.72–336.90)
H16.5C10	Ruike Biotechnology	Mouse	Conformational	392.19 (60.04–1381.00)

IC_50_ neutralizing activity is defined as the antibody concentration required to block viral entry by 50%. Values in parentheses are 95% confidence limits.

### Binding affinity and kinetics of a panel of HPV16 mAbs

Previous site-directed mutagenesis study showed that mAbs H16.25F12, H16.26H7, and H16.4G12—among the top six nAbs in the above-mentioned panel—shared some key epitope amino acids, suggesting that their binding regions are overlapping^12^. We then selected four representative mAbs, including H16.001, H16.8A9, H16.V5, and H16.4G12, for further binding study. Through SPR assay, as shown in Table 2 and Supplementary Fig. 1, H16.001 showed the highest affinity against HPV16 L1 pentamer as *K*_D_ of 4.22 × 10^−11^ M, while H16.8A9, H16.V5, and H16.4G12 possessed relatively high affinities of *K*_D_ with 1.18 × 10^−10^, 2.89 × 10^−9^, and 1.37 × 10^−9^ M, respectively, although they are lower than that of H16.001. Notably, H16.001 demonstrated the binding affinity as high as 67-fold than H16.V5 that was used in characterization of Gardasil^13^.

**Table 2:** Summary of the SPR measurements for four mAbs (H16.001, H16.8A9, H16.V5, and H16.4G12).

Antigen	Sample	*k*_a_, ×10^5^ [M^−1^ s^−1^]	*k*_d_, ×10^−5^ [s^−1^]	*K*_D_ [nM]
HPV16 pentamer	H16.001	2.37	<1	<0.042
	H16.8A9	1.93	2.28	0.118
	H16.V5	2.49	72	2.89
	H16.4G12	19.2	263	1.37

The tabulated dissociation constant (*K*_D_), association rate constant (*k*_a_), and dissociation rate constant (*k*_d_) were calculated using the global fit and the 1:1 binding model across 6 concentrations (10, 5, 2.5, 1.25, 0.625, and 0 nM) for HPV16 pentamer.

### Reactivity of mAbs to HPV16 VLPs from various expression systems

**Fig. 2:**
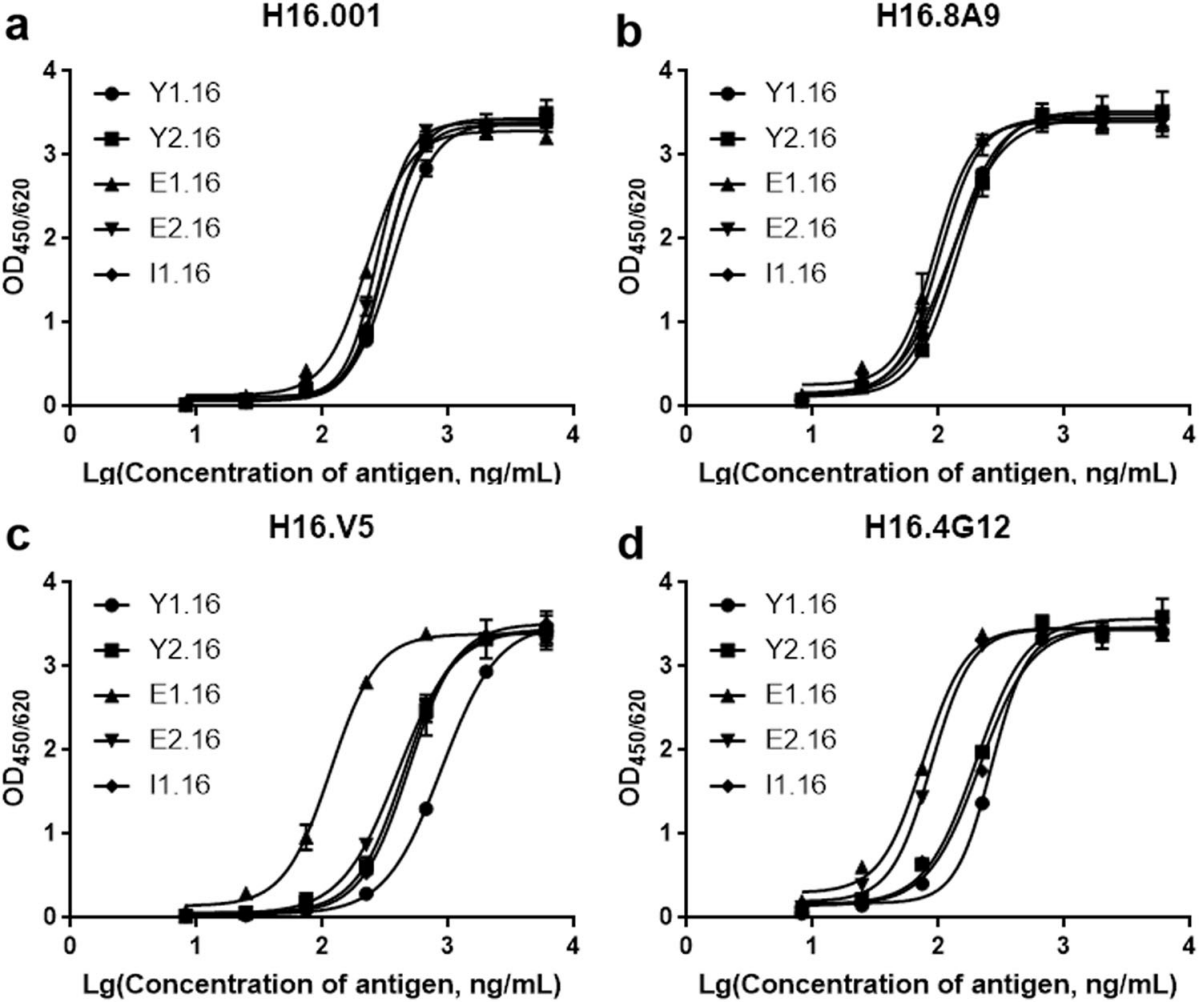
Binding profiles of four mAbs to HPV16 L1 VLPs derived from different expression systems. H16.001 and HRP-H16.001 (**a**), H16.8A9 and HRP-H16.8A9 (**b**), H16.V5 and HRP-H16.V5 (**c**), and H16.4G12 and HRP-H16.4G12 (**d**) were, respectively, used as capture antibody and detection antibody. The absorbance values were indicated on the *Y*-axis, and antigen concentrations were indicated on the *X*-axis. Each data point was the mean of two separate experiments. Y1.16, and Y2.16 were derived from yeast expression system. E1.16 and E2.16 were derived from *E. coli* expression system. I1.16 was derived from insect cell expression system.

To develop a common IVRP test for HPV16 VLPs derived from different expression systems (Table 3), we sought to identify some mAbs affordable to recognize various source VLPs comparably. The VLP samples Y1.16, and Y2.16 were produced in yeast expression system, E1.16, and E2.16 from *Escherichia coli* expression system, and I1.16 from insect cell expression system. Interestingly, H16.001 and H16.8A9 reacted with all sorts of HPV16 VLPs comparably as manifested by almost overlapping binding response curves for five HPV16 VLP samples, indicating that H16.001 and H16.8A9 reactivities are tolerant and irrespective of the expression systems that generated the HPV16 VLPs, whereas H16.V5 reacted the best with *E. coli*-derived E1.16 and worst with insect cell-derived I1.16. H16.4G12 bound with *E. coli*-derived VLPs (E1.16 and E2.16) preferably (Fig. 2). The commonality of H16.001 or H16.8A9 across distinct expression systems for VLP generation suggested its potential for IVRP test development used in vaccine regulation practice.

**Table 3:** Characterization of bulk HPV16 L1 VLPs.

HPV16 L1 VLPs	Property	Donator (vaccine manufacturers)	Expression system
Y1.16	Production	Ruike Biotechnology	Yeast
Y2.16	Production	Bowei Biotechnology	Yeast
E1.16	Production	Beijing Health Guard Biotechnology	*E. coli*
E2.16	Production	Innovax Biotech	*E. coli*
I1.16	Production	Sino Biological Inc.	Insect cell

### Immunodominance in anti-HPV16 sera of mAbs

**Fig. 3:**
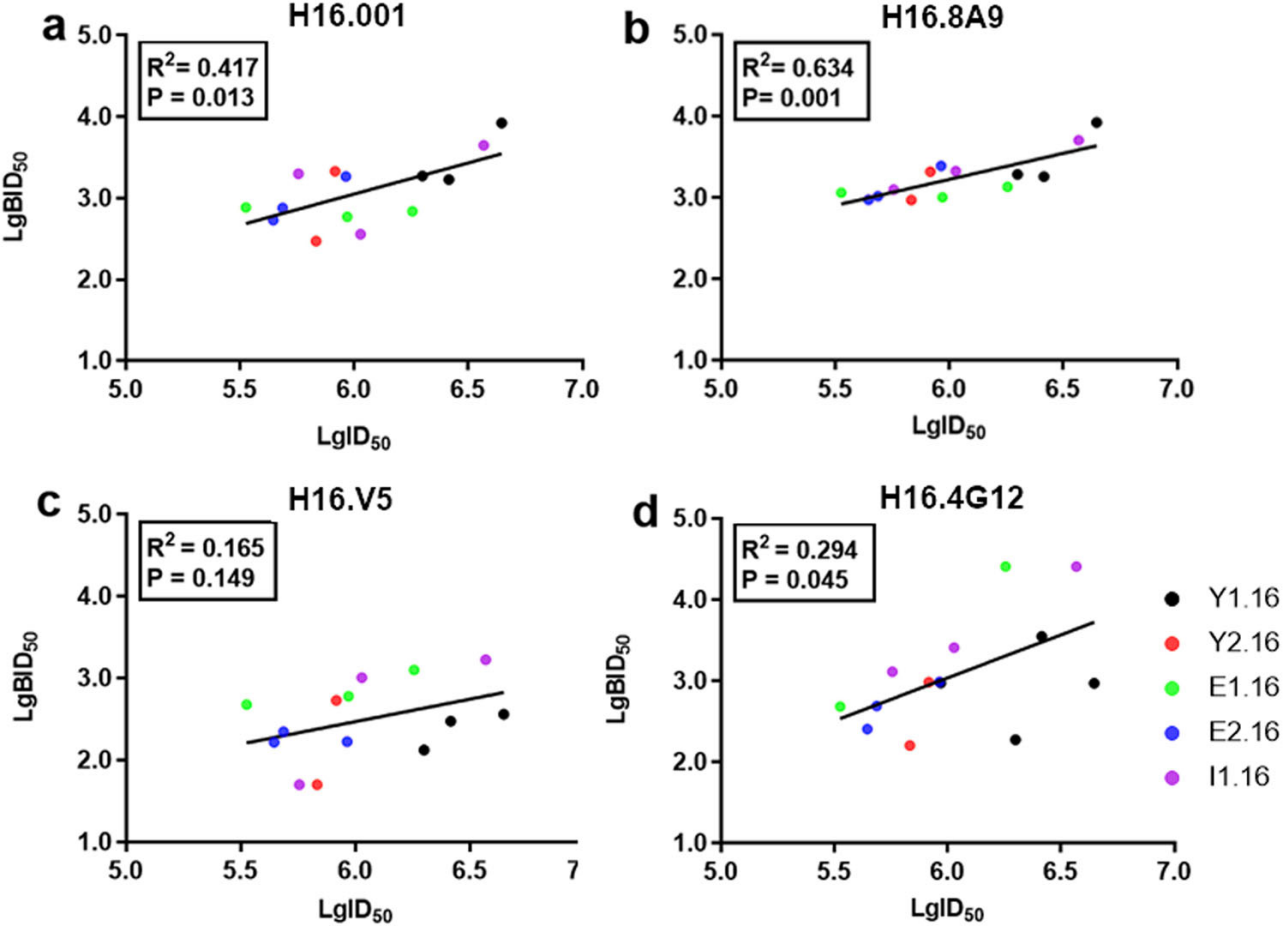
Correlation between the inhibition ratio by mAbs and neutralization titer of the vaccinated guinea pig sera. Correlation between BID_50_ and ID_50_ for H16.001 (**a**), H16.8A9 (**b**), H16.V5 (**c**), and H16.4G12 (**d**). ID_50_ was defined as the serum dilution required to block viral entry by 50%. BID_50_ was defined as the serum dilution required to block specific mAb binding by 50%. The black dots represent sera from guinea pigs vaccinated with Y1.16 VLPs produced by yeast expression system, the red ones represent sera with Y2.16 by yeast expression system, the green ones represent sera with E1.16 by *E. coli* expression system, the blue ones represent sera with E2.16 by *E. coli* expression system, and the purple ones represent sera with I1.16 by insect cell expression system. The comparison was assessed using the calculated Pearson coefficient of correlation, and the statistical analysis was performed using GraphPad Prism 7.

Immunodominance analysis for an mAb by competition ELISA (c/ELISA) or blocking ELISA (b/ELISA) usually reflect on the major neutralization epitope that generates corresponding antibodies in the anti-sera. We then measured the blocking ratio of the 4 mAbs against 14 guinea pig sera (Supplementary Table 1) from animals vaccinated with preparations of VLPs produced from 5 different manufacturing processes using yeast, *E. coli*, or insect cell expression through c/ELISA. BID_50_ value (a serum dilution titer capable of blocking mAb from binding to HPV VLPs by 50%) was used to quantify the dominance of specified mAb. mAb H16.001 had BID_50_ ranging from 295 to 8356, from 935 to 8356 for H16.8A9, from 100 to 1673 for H16.V5, and from 184 to 25,586 for H16.4G12 as blocking by the guinea pig serum samples in c/ELISA. Meanwhile, the neutralization titers (denoted as 50% maximal dilution concentration (ID_50_) value) for the sera were measured by PBNA and ranged from 442,588 to 4,425,884. Then correlation between BID_50_ and ID_50_ for each serum sample was plotted for the 4 mAbs and analyzed by linear regression fitting; H16.001 and H16.8A9 showed significant correlation between BID_50_ and ID_50_ as relatedness coefficient values of 0.417 (*P* < 0.05) and 0.634 (*P* < 0.001), respectively (Fig. 3a, b), H16.4G12 showed lower linear correlation with relatedness coefficient value of 0.294 (*P* < 0.05; Fig. 3d), whereas H16.V5 had no significant correlation (Fig. 3c). These results indicated that H16.001 and H16.8A9 were more immunodominant in vaccinated guinea pig sera with regard to neutralization titer.

**Fig. 4:**
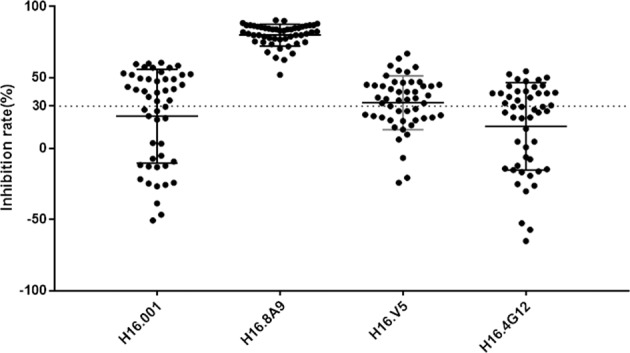
Inhibition ratios of the four mAbs against the 50 vaccinated human sera. VLP ELISA wells were reacted with surplus amounts of competing mAb before addition of human sera, and the inhibition ratios were then calculated. The human sera were obtained from individuals vaccinated with a yeast-derived VLP16/18 vaccine under clinical development (ClinicalTrials.gov ID:2011L01085). Each dot represents one serum sample (*n* = 50).

Next, we explored the blocking ratio of the 4 mAbs against HPV16 reactive human sera (Supplementary Table 1, *N* = 50) by b/ELISA in which HPV16 VLPs were premixed with mAb and then reacted with human sera. Notably, the human sera were obtained from individuals vaccinated with a yeast-derived VLP16/18 vaccine under clinical development (ClinicalTrials.gov ID:2011L01085). As shown in Fig. 4, H16.001 reduced 33–60% reactivities against HPV16 VLPs for 27 human sera, 12 of which were inhibited by >50%; H16.8A9 showed the most inhibition ratio for all the 50 human vaccinated sera as 52–91%, demonstrating that H16.8A9 epitope was immunogenic in the vaccine-induced humoral response in human; H16.V5 reduced 32–67% reactivities against HPV16 VLPs for 29 human sera, 7 of which were inhibited by >50%; H16.4G12 reduced 31–55% reactivities against HPV16 VLPs for 21 human sera, 3 of which were inhibited by >50%. Taken together, H16.001 and H16.8A9 identified some immunodominant neutralization epitope and their binding to HPV16 VLPs seemed to be irrespective of the expression systems that generated the VLPs.

### Cryo-electron microscopic (cryo-EM) structure of HPV16 PsV in complex with Fab H16.001

**Fig. 5:**
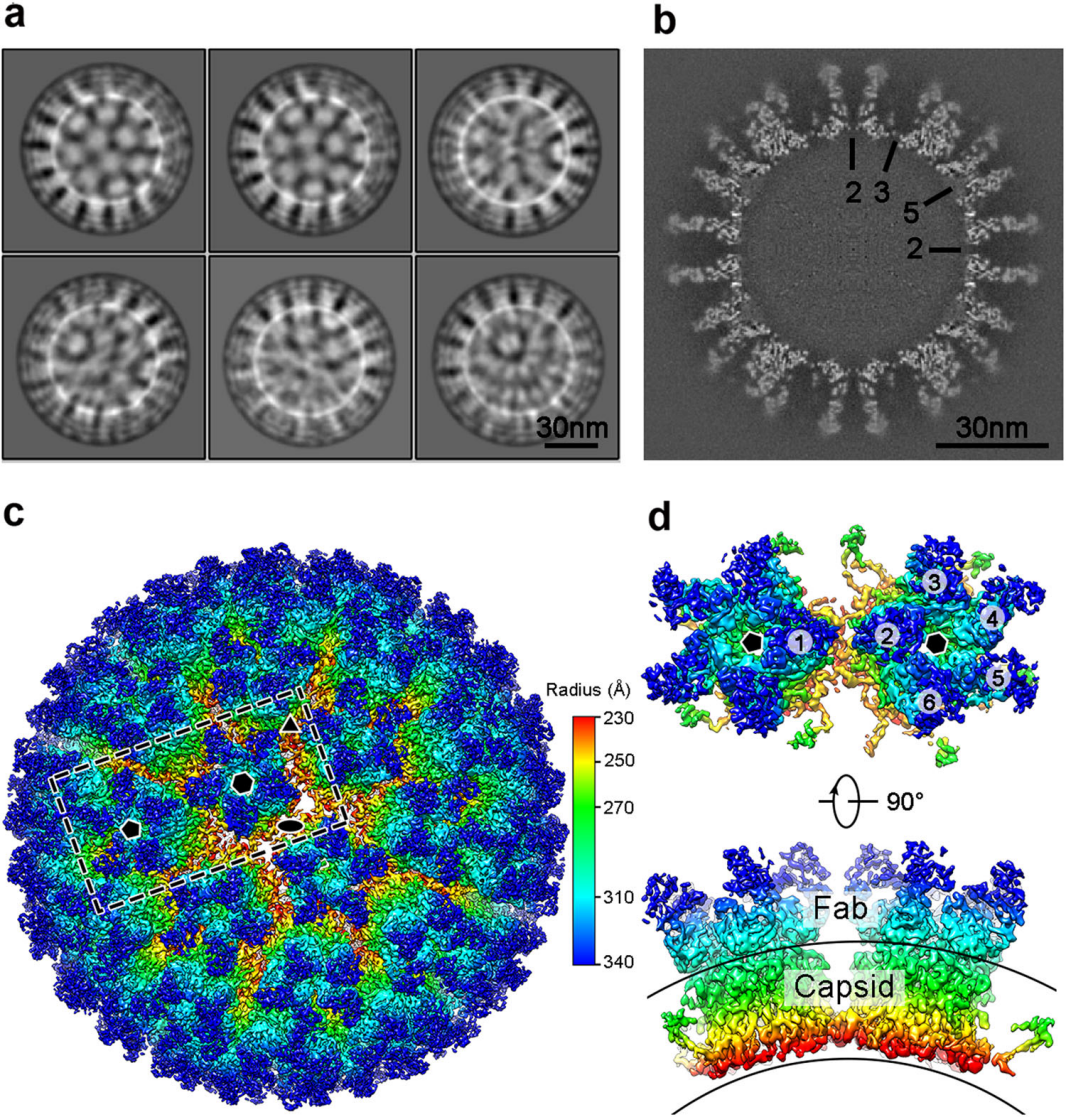
Cryo-EM structure reconstruction of HPV16 in complex with Fab H16.001. (**a**) Representative 2D classification averages of the immune-complex H16-001. (**b)** Central section of density map of the immune-complex H16-001 (on the twofold axis, with symmetry axes indicated in black). Scale bar = 30 nm. **c** The iso-contoured view of cryo-EM density map of the immune-complex H16-001 (radially colored) is shown along the icosahedral twofold axes. Icosahedral twofold, threefold, and fivefold axes are indicated by black symbols. (**d**) Closed-up view of the boxed area in (**c**). The number indicates the six L1 monomers in an icosahedral asymmetric unit. The pentagon and hexagon symbols represent 5-coordinated and 6-coornidated pentamer, respectively.

As HPV16 VLPs were shown to be extremely heterogeneous, it was not suitable for high-resolution structure determination. To elucidate the potentially binding modality of HPV16.001 that preferably suits for vaccine characterization, we prepared the immune complex (termed HPV-001) by mixing HPV16 PsV with Fab H16.001 at a molar ratio of 1:1.5 for cryo-EM analysis. The cryo-EM micrographs and two-dimensional (2D) classifications analysis revealed that the HPV-001 immune complex have a generally homogenous population with a diameter of ~700 Å. Further, the clearly observed protrusions demonstrated the Fab binding to the viral particle (Fig. 5a and Supplementary Fig. 2a). Finally, we selected a total of 9162 particles to reconstruct the immune-complex structure imposing icosahedral symmetry with cisTEM^14^. The resolution of the final map was determined at 4.41 Å using the “gold” standard Fourier Shell Correlation (FSC) = 0.143 criterion (Supplementary Fig. 2b)^15^. The central section of HPV-001 density map shows the high quality of the reconstructions (Fig. 5b). High-resolution cryo-EM density map in radial color revealed H16.001 Fabs could bind both 5-coordinated and 6-coordinated pentamer in HPV16 PsV, and thus a total of 360 Fabs engage to a *T* = 7 HPV16 capsid. (Fig. 5c, d and Supplementary Movie 1). Further, as the immune-complex structures reported in H16.V5, the Fab-binding occupancies were various within one icosahedral asymmetric unit (loci −1 to −6), especially in locations −1 and −2, which could be a result of steric hindrance (Supplementary Fig. 3)^16^. Unlike H16.V5-binding pattern, H16.001 Fab densities in 5-coordinated pentamer (location −1) did not overlap with that in 6-coordinated pentamer (loci −2 to −6) (Fig. 5d), which enable us to reconstruct the Fab density map more confidently.

**Fig. 6:**
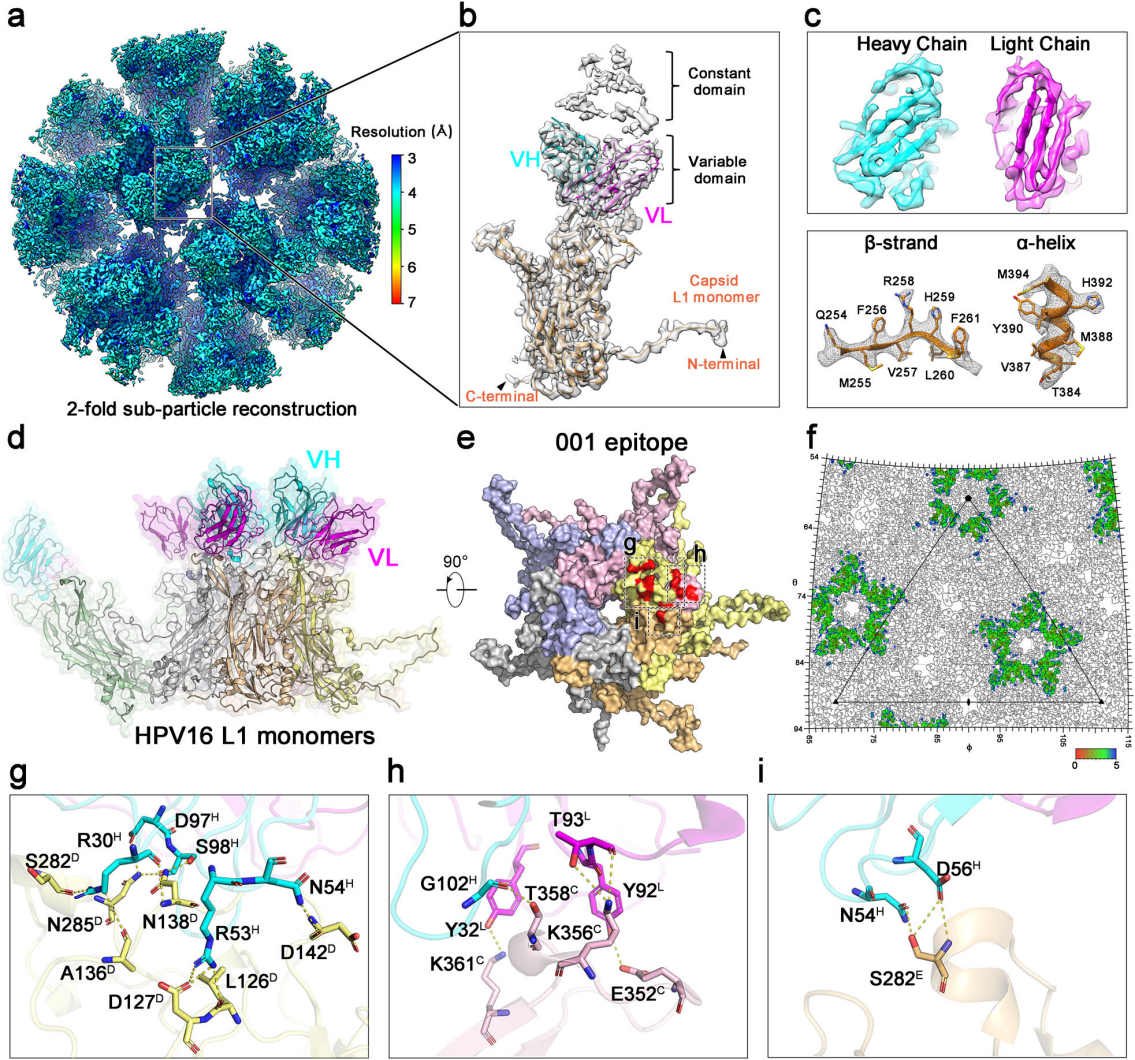
Subparticle reconstruction and interaction analysis of HPV16:001 immune complex. **(a)** The cryo-EM structures of twofold axis subparticle region was surface colored according to the local resolution ranging from 3 to 7 Å. (**b)** Close-up view of the L1 monomer and the bound Fab density map, which was fitting with the corresponding models in ribbon diagram excluding the Fab constant domain. (**c)** Electron density maps from the segment of Fab and capsid. (**d)** One icosahedral asymmetric unit of H16-001 structure with a monomer and its bound Fab. (**e)** The footprints of Fab H16.001, of which the dashed box indicated the different L1 monomers with Fab H16.001 interaction. (**f)** 2D projection of H16.001 footprints on HPV16 surface produced by RIVEM. The footprints are highlighted and colored according to the distance of particle surface to Fab from red to blue. One icosahedral asymmetric of HPV16 is shown in a black triangle. (**g**–**i)** Close-up views of the chain-D (**g**), chain-C (**h**), and chain-E (**i**) with capsid interaction. The potential hydrogen bonding and salt bridges in interaction sites were labeled and marked by yellow dashed lines. The different chains in one icosahedral asymmetric unit were colored in the same scheme as follows: chain-A in pale green, chain-B in light chain, chain-C in light pink, chain-D in pale yellow, chain-E in light orange, chain-F in gray, heavy chain in cyan, and light chain in magenta.

However, such 4.41 Å resolution was still insufficient for model building, which might have resulted from the sample heterogeneity and the flexibility of the virion or the Fab. To conquer these issues and improve the resolution, we extracted the twofold axis region comprising the viral asymmetric unit with a diameter of 451.2 Å as subparticle for further refinement using the localized reconstruction procedure^17^. The resolution was finally determined to 3.43 Å by “gold” standard FSC = 0.143 criterion (Fig. 6a and Supplementary Fig. 2a). The backbone of the polypeptide, as well as amino acid side chains, can be clearly traced in the capsid and Fab variable domain (Fig. 6b, c), enabling us to build the majority of the capsid protein and the variable domain of H16.001 Fab but excluding the constant domain that was disordered density as shown in Fig. 6b. In an asymmetric unit, each fab binds across three L1 monomers (here termed chain-C, chain-D, and chain-E) with a total buried surface area of ~1183 Å^2^, with about 73% (~867 Å^2^) buried by the heavy chain (Fig. 6d–f). Of note, chain-D occupy a dominant role in Fab–capsid interaction with a buried surface of ~762 Å^2^ (64%), while for chain-C and chain-E it is only ~315 Å^2^ (27%) and ~106 Å^2^ (9%), respectively (Fig. 6d–f). Nineteen hydrogen bonds and salt bridges, as well as several van der Waals interactions, establish the elaborated interaction network, which were majorly donated from the DE and FG loops from chain-D, HI loop from chain-C, and FG loop from chain-E (Fig. 6g–i and Supplementary Table 2). Among these interaction sites, the residues D127 from chain-D and S282 from chain-E forming three hydrogen bonds and salt bridges indicated them as critical role in H16.001 Fab binding to the capsid. As compared with other reported structures of HPV-Fab immune complex (HPV-V5 and HPV-U4), the full-occupied binding to whole capsid without any steric hindrance between neighboring Fabs is assumed to be its preference for vaccine characterization such as IVRP, in particular has tolerance upon various expression systems being applied in VLPs production.

## Discussion

Here we present comparable biochemical and virological studies of a panel of HPV16 nAbs. Based on our study, H16.001 had the strongest neutralizing activity with 0.40 ng/ml IC_50_ value (Fig. 1). Meanwhile, H16.001 had the highest binding affinity with a 4.22 nM *K*_D_ value (Table 2 and Supplementary Fig. 1). It seemed that the top neutralizer tended to be the top binder, which was consistent with a previous study where 8H7 had the smallest NC_50_ (neutralization) and EC_50_ (affinity) values^18^. Notably, our previous study showed that the neutralization efficacy of H16.001 was not significantly affected by all the 31 naturally occurring HPV16 variants^12^, indicating a broader protection of H16.001. These results suggested that H16.001 was an mAb with more potent neutralizing activity, higher affinity, and broader protection and can be tailored for evaluating the integrity and antigenicity of HPV16 vaccines from different expression systems.

Currently, the IVRP assay, a sandwich-type immunoassay, has been developed as an alternative to the mouse potency assay, which was usually used for lot release of the HPV prophylactic vaccines. And the relative antigenicity measured by the IVRP assay is demonstrated to be a good predictor of in vivo potency^13^. However, the vaccine manufacturers apply different expression systems to produce their vaccine and develop the IVRP assays based on their own mAbs to evaluate the vaccine, which makes the results incommensurable among different manufacturers’ vaccines. So an mAb that can identically recognize various HPV16 L1 VLPs from different expression systems is urgently needed. Fortunately, our study found that the binding response curves of H16.001 to HPV16 L1 VLPs derived from three expression systems were almost identical, which indicated that H16.001 could identically bind the various HPV16 L1 VLPs (Fig. 2). Structural study showed that five H16.001 Fabs bound to one pentamer with low steric hindrance for neighboring H16.001 Fabs (Fig. 5c), which may partially explain why H16.001 can show same sensitivities to various HPV16 L1 VLPs.

H16.001 and H16.8A9 recognize more immunodominant epitopes than others in HPV vaccine recipients. The PBNA was used to determine the functional activity of total neutralizing antibodies to inhibit a PsV infection, while epitope-specific c/ELISA assay was used to define the neutralizing antibody titers directed to a certain epitope on the viral capsid. It is reasonable to consider that more immunodominant epitope resulted in better correlation between the two methodologies. Our epitope-specific c/ELISA assay showed that antibodies which recognized the same epitope as H16.001 or H16.8A9 correlated better with total neutralizing antibodies in vaccinated guinea pig sera than others (Fig. 3). Meanwhile, 12 and 50 vaccinated human sera were strongly inhibited by mAbs H16.001 and H16.8A9, respectively (Fig. 4). These results suggested that H16.001 or H16.8A9 represented the majority of neutralizing antibodies in HPV16 vaccine recipients, indicating that two antigenic sites were immunodominant in the humoral response to HPV16 L1 VLPs vaccination: one recognized by the H16.001, and the other by H16.8A9.

To date, there are several reported cryo-EM structures of immune complex containing HPV PsV, wherein the highest resolution was only at 4.7 Å and could not afford a confident model building at near atomic level^16,19–21^. We circumvented the resolution bottleneck over 4 Å resolution for HPV capsid immune complex through new emerging subparticle approach. The subparticle extraction from the large H16-001 immune complex with a diameter of ~700 Å allowed local reconstruction and accurate determination of defocus value and eventually improved the resolution from 4.41 Å to 3.43 Å. The approach will determine more structures of immune complexes in expedient and high-resolution manner and definitely accelerate the anti-virus drug and vaccine design. Furthermore, the H16-001 cryo-EM structure revealed a distinctive antibody-binding pattern as compared to mAbs H16.V5 and H16.U4. H16.V5 Fab preferentially binds with the hexavalent capsomer, and H16.U4 Fab preferentially binds with the pentavalent capsomer^16^, whereas H16.001 Fab can engage both hexavalent and pentavalent capsomeres of HPV capsid (Figs. 5 and 6a, d). Such unique binding mode might be associated with its high potent neutralizing activity over other mAbs. Interaction analysis revealed that H16.001 Fab-binding footprint is mainly located at DE, FG, and HI loops of HPV16 L1, overlapping with those epitopes of mAbs H16.V5, 1A, 14J, and 263A2^16,21^. Intriguingly, these five mAbs recognize three common sites, at S282, N285, and T358, which might be associated with the cellular attachment and entry during HPV16 infection.

Taken together, we identified a rabbit monoclonal antibody H16.001 recognizing a potent neutralizing, high affinity, immunodominant, and broad protection epitope. Importantly, the unique binding modality of this antibody to the HPV16 capsid protein may contribute to be used for in vitro assays to evaluate all HPV vaccine from different expression systems. Further studies need to be conducted to explore the use of H16.001 in practical quality assessment work for HPV vaccines, such as developing a common potency assay.

## Methods

### Plasmids, mAbs, bulk HPV16 L1 VLP products, HPV16 pentamers, and post-vaccinated human serum samples

The HPV16 packaging plasmid (p16LLw) containing the codon-optimized L1 gene (HPV16 isolate 114K) and L2 gene (isolate 114B) was kindly provided by Dr. John T. Schiller (National Cancer Institute, Bethesda, MD)^4,22^.

As shown in Table 1, 13 neutralizing mAbs against HPV16 were kindly provided by 5 vaccine manufacturers, with H16.5C10 by Ruike Biotechnology (Jiangsu, China); H16.V5 and horseradish peroxidase (HRP)-H16.V5 by Bowei Biotechnology (Shanghai, China); H16.4G12, H16.6C7, H16.26H7, H16.25F12, H16.25H8, H16.22G5, H16.17B10, H16.20G3, and HRP-H16.4G12 by Beijing Health Guard Biotechnology (Beijing, China); H16.8A9, H16.1D12, and HRP-H16.8A9 by Innovax Biotech Co., Ltd. (Xiamen, China); and H16.001 and HRP-H16.001 by Sino Biological Inc. (Beijing, China).

As shown in Table 3, five kinds of bulk HPV16 L1 VLP products were also kindly provided by the aforementioned vaccine manufacturers, with Y1.16 from Ruike Biotechnology, Y2.16 from Bowei Biotechnology, E1.16 from Beijing Health Guard Biotechnology, E2.16 from Innovax Biotech Co., Ltd., and I1.16 from Sino Biological Inc. Notably, Y1.16 and Y2.16 were produced in yeast expression system, E1.16 and E2.16 from *E. coli* expression system, and I1.16 from insect cell expression system.

HPV16 pentamers were kindly provided by Innovax Biotech Co., Ltd. Fifty post-vaccinated human serum samples were kindly provided by the vaccine manufacturer Shanghai Zerun Biotechnology (Shanghai, China), which were collected from a phase I clinical trial of a bivalent HPV16/18 vaccine, produced in Pichia Pastoris (ClinicalTrials.gov ID:2011L01085). Vaccines were administered according to their recommended three-dose vaccination schedules (months 0, 2, and 6). Serum samples were collected 7 months after the first immunization.

### Fab preparation

To obtain H16.001 Fab, the mAb H16.001 was digested in the mixture solution containing 1‰ (w/w) papain, 20mM L-Cysteine, and 50 mM ethylenediamine tetraacetic acid at 37 °C. Following 12-h incubation, 30 mM iodoacetamide was added to stop the digestion. The resulting Fab fragment was then purified by diethylethanolamine column (TOSOH, Japan) chromatography.

### Generation and purification of HPV16 PsVs

293FT cells were grown in complete Dulbecco’s modified Eagle’s medium containing 10% fetal bovine serum, 2% HEPES, 1% nonessential amino acids, and 1% penicillin/streptomycin. The reporter plasmid pcDNA3.1-EGFP was constructed by inserting an EGFP reporter gene into the pcDNA3.1 vector (Invitrogen, Carlsbad, CA). HPV16 L1L2 PsVs were generated and titrated as described previously^4,23,24^. The PsV titers were defined as 50% tissue culture infective dose, calculated with the Reed–Muench method^25^. For structure determination, PsV particles were purified by sucrose density gradient and subsequent CsCl density gradient ultracentrifugation^26^.

### Sera from immunized guinea pigs with HPV vaccines

The animal study was approved by the Institutional Animal Care and Use Committee of National Institutes for Food and Drug Control. All animals were housed in accordance with relevant guidelines. Five samples of HPV16 VLPs, which were kindly provided by Ruike Biotechnology, Bowei Biotechnology, Health Guard Biotechnology, Innovax Biotech Co., Ltd., and Sino Biological Inc., were mixed with aluminum hydroxide adjuvant, respectively. Ten micrograms of the aforementioned adjuvant-adsorbed HPV16 VLPs were delivered intramuscularly for each guinea pig (200–220 g, female) and three guinea pigs were immunized with each of the vaccines. At the same time, the aluminum hydroxide adjuvant was inoculated into three guinea pigs intramuscularly as a control. Immunization was repeated twice at 3-week intervals, and sera were obtained 3 weeks after the second immunization. All serum samples were inactivated before the first use.

### PsV-based neutralization assay

Neutralization properties of the mAbs or sera were assessed as described^23,24,27,28^. Briefly, the PsVs were mixed for 1 h at 4 °C with medium, serial dilutions of mAbs or sera. 293FT cells were plated 4–6 h before the mixtures were added with 1.5 × 10^4^cells/100 μL/well. The aforementioned combinations were transferred into cell culture plates preseeded with 293FT cells and incubated for 68–72 h. After incubation, the numbers of fluorospots were counted with an ImmunoSpot reader. The mAbs and serum neutralization titers were defined as the IC_50_ and ID_50_, respectively, and calculated with the Reed–Muench method^25^.

### SPR binding assay

Affinity measurement was conducted at 25 °C on a BiacoreT200 instrument (GE Healthcare, Uppsala, Sweden). mAb (25 μg/mL) was directly immobilized to the surface of a CM5 sensor chip via covalent amine coupling for 60 s at a flow rate of 5 μL/min. HPV16 pentamer was initially diluted as 20 nM and then serial diluted to five concentrations (10, 5, 2.5, 1.25, and 0.625 nM) in 250 μL by using HBS-EP+ buffer, pH7.4 (GE Healthcare, Uppsala, Sweden). Then HPV16 pentamer analyte with each concentration was loaded onto sensor chip to carry out an association step with mAb ligand for 120 s for determining their relative on-rates; this was followed by a dissociation step for 600 s for determining their relative off-rates. Ten millimoles of glycine-HCl (pH 1.5) was used as regeneration buffer at a flow rate of 10 μL/min for 30 s. The SPR curves were recorded and processed with the subtraction of buffer reference and then evaluated using the BIA evaluation software.

### IVRP test

Bulk HPV16 L1 VLP product concentrations were analyzed at the Innovax Biotech Co., Ltd. (Xiamen, China) using a TCA-Lowry assay. And protein concentration was determined relative to a bovine serum albumin (BSA) standard curve.

The IVRP assay is a sandwich immunoassay that uses the same mAb for capture and detection. The assay was performed by first coating Immulon96-well microplate (Thermo Scientific) with the mAb diluted in phosphate-buffered saline (PBS) at a concentration of 2 μg/mL. These antibody-coated plates were referred to as assay plates. The bulk HPV16 L1 VLP products were prepared in 0.5% BSA-PBST at 7 concentrations of 8.2, 24.7, 74.1, 222, 667, 2000, and 6000 ng/mL, and 100 μL of each dilution was added to the assay plate. The plate was allowed to incubate for 1 h at 37 °C and was washed before 100 μL of the diluted HRP-mAb at a concentration of 2 μg/mL was added. All loaded plates were subsequently incubated at 37 °C for 1 h, washed three times with PBST, and developed with TMB. The reaction was stopped with 2 N H_2_SO_4_ and the optical density (OD) of each well of the plate was determined on a microplate reader at 450/620 nm.

### The immunized guinea pig sera tested by competition ELISA using different mAbs

Immulon96-well microplates (Thermo Scientific) were coated with 100 μL of Y1.16 (2 μg/mL) and incubated overnight at 4 °C. Plates were incubated with 100 μL of guinea pig sera for 1 h at 37 °C. Guinea pig sera were initially diluted at 1:100 and serially diluted twofold in 0.5% BSA-PBST across the plate in duplicates. After incubation, plates were washed with PBST three times and incubated with specific HRP-mAb. After 1-h incubation at 37 °C, TMB and 2 N H_2_SO_4_ were added as aforementioned above, and the OD was read at 450/620 nm. BID_50_ blocking activity is defined above in the “Results” section.

### The immunized human sera tested by blocking ELISA using different mAbs

The proportion of vaccinated human sera directed against different HPV16 mAbs was determined by a blocking ELISA, in which Y1.16 was incubated with saturating levels of mAbs prior to the addition of vaccinated sera, and residual binding of human immunoglobulin G (IgG) was detected using HRP-anti-human IgG. Saturating mAbs (100 μL, 5 μg/mL in 0.5% BSA-PBST) were added to VLP-coated plates for 1 h at 37 °C, followed by the addition of 100 μL of vaccinated serum diluted 1:6000 in BSA-PBST for a further 1 h. Dilutions of vaccinated sera were chosen in order to give an ELISA result of approximately 1.0 OD units. Plates were then washed with PBST, and residual binding of human IgG was detected using HRP-conjugated sheep anti-human IgG. To quantitate the residual binding of human IgG, any difference in the level of human IgG binding between the control (0.5% BSA-PBST) and specific mAbs is therefore a measure of the proportion of total HPV16-specific IgG directed against epitopes, which are altered or blocked by binding of the specific mAb. Notably, 30% was used as a cutoff, and sera reducing the photometer extinction ≥30% were defined as positive.

### Cryo-EM sample preparation and data acquisition

HPV-001 immune complex was prepared by the mixture of HPV16 PsV with Fab H16.001 with a molar ratio of 540 Fabs to every HPV16 viral particle incubating at 37 °C for 30 min. Aliquots (3 μL) of the immune-complex sample were deposited onto the glow-discharged holey carbon Quantifoil Cu grids (R2/2, 200 mesh, Quantifoil Micro Tools). The grids were blotted for 6 s by filter paper at 100% humidity and 4 °C using Vitrobot Mark IV and then plunged-frozen into liquid ethane. Prepared grids were examined using 300 kV Tecnai F30 microscope (Thermo Scientific) equipped with a Falcon II direct electron detector. The cryo-EM data were collected at a nominal magnification of ×93,000 in 17-frame movie mode, yielding a pixel size of 1.128 Å. The total exposure dose is 30 e− Å^−2^ and exposure time is 1 s.

### Cryo-EM data processing

Beam-induced motion correction was executed by MotionCor2^29^. Each micrograph contrast transfer function was estimated using Gctf^30^. A total of 12,034 particles (768-by-768 pixels) were semi-automatic boxed-out from 880 micrographs using e2boxer.py^31^ and subjected to reference-free 2D classification using Relion 3.0^32^. Nine thousand one hundred and sixty-two particles were selected for initial model generation and auto-refined to be 4.41 Å resolution according to the “gold” standard FSC = 0.143 criterion imposing icosahedral I2 symmetry using cisTEM^14^. For further improving the resolution and conquering the sample heterogeneity and defocus gradient, we applied the localized reconstruction method^17^ to extract the subparticles for refinement and reconstruction. Briefly, based on the orientation and center parameters of each particle image, which was calculated from the icosahedral symmetry refinement, we extracted the subparticles in a box size of 400 × 400 pixels centered at 290 Å from the center of the particle. This resulted in a total of 274,860 subparticles with their defocus values re-calculated from their locations in each viral particle. Such subparticles were then imported into cisTEM for manual local refinement imposing C2 symmetry, which were determined to be 3.43 Å resolution with FSC cutoff of 0.143. Local resolution variations were estimated using ResMap^33^.

### Model building and refinement

The initial model of H16.001 Fab was generated from homology modeling using the Accelrys Discovery Studio software^34^. Then we generated the initial template model (enclosing an icosahedral asymmetric unit) by docking the homology Fab models and HPV16 capsid structure (PDB code: 5KEP) into the density map using Chimera^35^. The template was further refined and rebuilt iteratively against the cryo-EM map using phenix. real_space_refine^36^ and Coot^37^. The refinement statistics of the final model is provided in Supplementary Table 3, as validated by MolProbity^38^. The footprint of Fab H16.001 was generated onto the 2D projection of the stereographic sphere using RIVEM^39^. Fab–capsid interaction analysis and buried surface area calculation were performed using the PISA server (www.ebi.ac.uk/pdbe/pisa). Structural figures were illustrated by PyMOL^40^ and Chimera^35^.

### Statistical analyses

Correlation between the inhibition ratio by mAbs and neutralization titer of the vaccinated guinea pig sera was determined using the calculated Pearson coefficient of correlation. All analyses were performed using the GraphPad Prism 7 software.

### Reporting summary

Further information on research design is available in the Nature Research Reporting Summary linked to this article.

## Supplementary information

Supplementary Movie 1

Supplementary Information

Reporting Summary Checklist

## Data Availability

The data that support the findings of this study are available from the corresponding author upon reasonable request. The cryo-EM density map and the corresponding atomic model have been deposited in the Electron Microscopy Data Bank (EMDB, http://www.ebi.ac.uk/pdbe/emdb/) and Protein Bank (PDB, https://www.ebi.ac.uk/pdbe/), respectively. The accession codes are: HPV:001 (EMDB: EMD-30415) and HPV:001 twofold sub-particle reconstruction (EMDB: EMDB-30414, PDB: 7CN2).
