# Supplementary material for: Structural characterization of a neutralizing mAb H16.001, a potent candidate for a common potency assay for various HPV16 VLPs
Source: NPJ Vaccines. 2020 Sep 23;5:89. doi: 10.1038/s41541-020-00236-w (PMC7511963; doi:10.1038/s41541-020-00236-w)
Supplement: Supplementary file 2 — Supplementary Information [file 41541_2020_236_MOESM2_ESM.pdf]

Supplementary Figure legends

Supplementary Figure 1. Binding kinetics of the four mAbs. H16.001 **(a)**, H16.8A9 **(b)**, H16.V5 **(c)**, and H16.4G12 **(d)** were immobilized as ligand, and HPV16 pentamers in the mobile phase as analytes.

Supplementary Figure 2. Cryo-EM micrographs and FSC curves of HPV-001 immune complex. (a) Representative raw cryo-EM images of HPV-001 immune complex samples. The scale bar is 45 nm. **(b)** The FSC curves of 3D reconstruction of HPV-001 imposing icosahedral symmetry (black line) and sub-particle reconstruction in 2-fold axes (red line).

Supplementary Figure 3. Cryo-EM structure reconstruction of HPV16 in complex with Fab H16.V5 (EMD-8243). (a) The iso-contoured view of cryo-EM density map of the immune-complex H16-V5 (radially colored) is shown along the icosahedral 2-fold axes. **(b)** Closed-up view of the boxed area in panel A. The number indicates the six L1 monomers in an icosahedral asymmetric unit, which were named as loci -1 to -6. Notably, the V5 Fab density in location-1 was weaker than other locations in one icosahedral asymmetric. Icosahedral 2-, 3- and 5-fold axes are indicated by black symbols. The pentagon and hexagon symbols represent 5-coordinated and 6-coornidated pentamer, respectively.

Supplementary Figure 1.

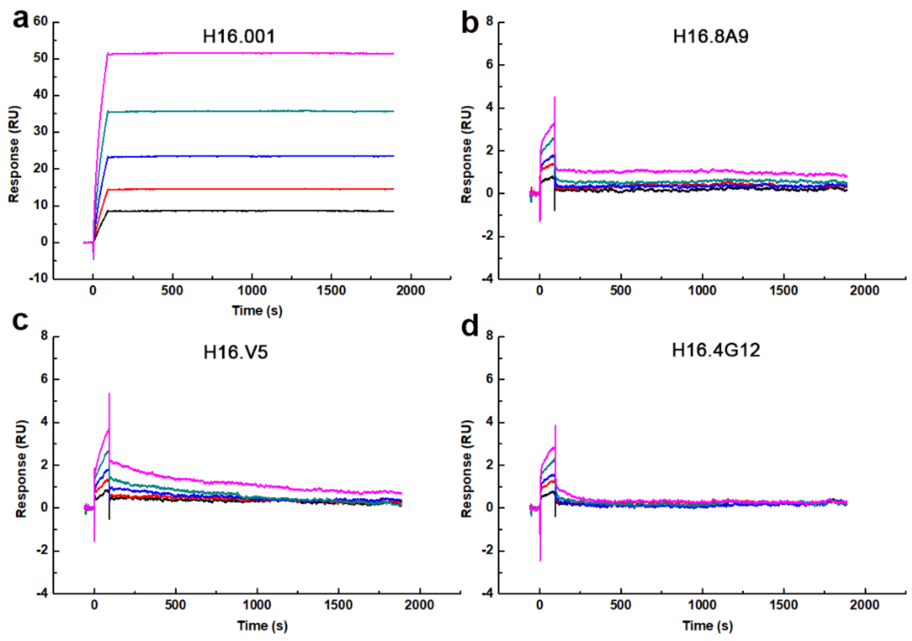

Supplementary Figure 2.

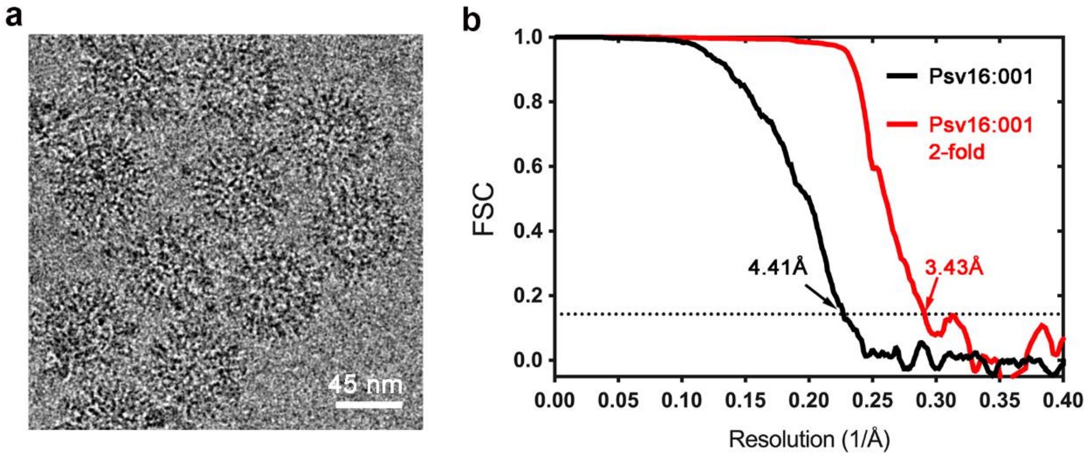

Supplementary Figure 3.

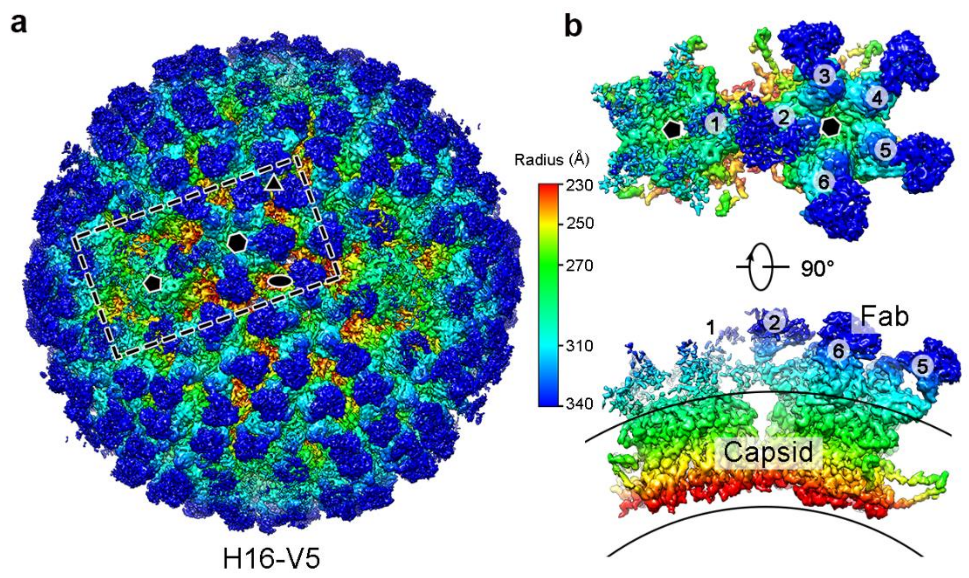

Supplementary Table 1. Neutralization titer of guinea pig sera and human sera immunized with HPV16 vaccine.

Guinea pig sera		Human sera			
No.	Neutralization titer	No.	Neutralization titer	No.	Neutralization titer
1	2600160	1	125287	26	36159
2	4425884	2	171214	27	203377
3	1990673	3	522565	28	36489
4	680769	4	127308	29	79702
5	824138	5	177332	30	56247
6	335738	6	143393	31	129538
7	933254	7	1679616	32	91950
8	1798871	8	764947	33	139720
9	920450	9	177558	34	83796
10	442588	10	124882	35	156981
11	486407	11	106014	36	7233
12	568853	12	178919	37	6059
13	1066596	13	86450	38	7694
14	3689776	14	32289	39	14478
		15	76247	40	18664
		16	127599	41	25995
		17	36228	42	2883
		18	28820	43	6553
		19	35202	44	12205
		20	28623	45	167107
		21	40490	46	10434
		22	96537	47	130966

23	33973	48	77397
24	79343	49	143895
25	140523	50	169406

Supplementary Table 2. Interaction sites between the HPV16 epitope and the H16.001 Fab.

H16.001's epitope on HPV16		H16.001 Fab residues		Distance (Å)
LEU126^{D}	(O)	ARG53^{H}	(NH2)	3.01
ASP127^{D}	(OD1)	ARG53^{H}	(NH2)	2.63
	(OD1)	ARG53^{H}	(NH2)	2.63*
	(OD1)	ARG53^{H}	(NH1)	3.28*
ALA136^{D}	(O)	ARG30^{H}	(NE)	3.90
ASN138^{D}	(N)	ARG30^{H}	(O)	3.50
	(ND2)	SER98^{H}	(OG)	2.49
ASP142^{D}	(O)	ASN54^{H}	(ND2)	3.84
SER282^{D}	(O)	ARG30^{H}	(NH2)	2.86
ASN285^{D}	(ND2)	ASP97^{H}	(OD1)	3.49
	(ND2)	SER98^{H}	(O)	2.66
SER282^{E}	(OG)	ASN54^{H}	(OD1)	3.30
	(N)	ASP56^{H}	(OD1)	3.47
	(OG)	ASP56^{H}	(OD1)	3.68
GLU352^{C}	(OE2)	TYR92^{L}	(OH)	2.88
LYS356^{C}	(NZ)	THR93^{L}	(O)	3.64
	(NZ)	THR93^{L}	(OG1)	3.85
THR358^{C}	(OG1)	GLY102^{H}	(O)	2.89
LYS361^{C}	(NZ)	TYR32^{L}	(OH)	3.17

* This contacting specifically refers to salt bridge.

Supplementary Table 3. Cryo-EM data collection and atomic model refinement statistics.

	HPV:001	HPV:001 2-fold sub-particle reconstruction
Data Collection and processing		
EM equipment	Tecnai F30	
Voltage (kV)	300	
Detector	Falcon II	
Pixel size (Å)	1.128	
Electron dose (e^-/$Å^2$)	39	
Defocus range (µm)	1.3-4.0	
Software	Relion 3.0, cisTEM, localized reconstruction	
Number of used Particles	9,162	274,860
Map Resolution (Å)	4.41	3.43
FSC threshold	0.143	0.143
Map sharpening B factor ($Å^2$)	-250	-150
Refinement	n/a	
Model composition		
Non-hydrogen atoms		32,841
Protein residues		4,233
Ligands		0
B factors ($Å^2$)		
Protein		-122.32
Ligand		0
R.m.s deviations		
Bond lengths (Å)		0.007
Bond angles (°)		1.292
Validation		
MolProbity score		2.47
Clashscore		8.80
Poor rotamers (%)		4.47
Ramachandran plot		
Favored (%)		91.52
Allowed (%)		7.91
Disallowed (%)		0.57

Supplementary Movie 1. Depicts the global reconstructed features of HPV-001 immune complex, and close-up view of the H16.001 binding region.
